# Supplementary material for: A Conductive Microcavity Created by Assembly of Carbon Nanotube Buckypapers for Developing Electrochemically Wired Enzyme Cascades
Source: Nanomaterials (Basel). 2024 Mar 20;14(6):545. doi: 10.3390/nano14060545 (PMC10976184; doi:10.3390/nano14060545)
Supplement: Supplementary file 1 [file nanomaterials-14-00545-s001.zip › nanomaterials-2766079-supplementary.pdf]

nanomaterials

Supporting Materials

A Conductive Microcavity Created by Assembly of Carbon Nanotube Buckypapers for Developing Electrochemically Wired Enzyme Cascades

Itthipon Jeerapan [1,2,*], Yannig Nedellec [3] and Serge Cosnier [3,4,5,*]

[1] Division of Physical Science and Center of Excellence for Innovation in Chemistry, Faculty of Science, Prince of Songkla University, Hat Yai 90110, Thailand
[2] Center of Excellence for Trace Analysis and Biosensor, Prince of Songkla University, Hat Yai 90110, Thailand
[3] Université Grenoble Alpes, Département de Chimie Moléculaire, UFR de chimie et de biologie, UMR 5250, F-38000 Grenoble, France; CNRS, DCM UMR 5250, F-38000 Grenoble, France
[4] Centre for Organic and Nanohybrid Electronics, Silesian University of Technology, Konarskiego 22B, 44-100 Gliwice, Poland
[5] Department of Physical Chemistry and Technology of Polymers, Silesian University of Technology, M. Strzody 9, 44-100 Gliwice, Poland
* Correspondence: itthipon.j@psu.ac.th (I.J.); serge.cosnier@univ-grenoble-alpes.fr (S.C.)
ORCID of Itthipon Jeerapan: https://orcid.org/0000-0001-8016-6411
ORCID of Serge Cosnier: https://orcid.org/0000-0002-8290-4374

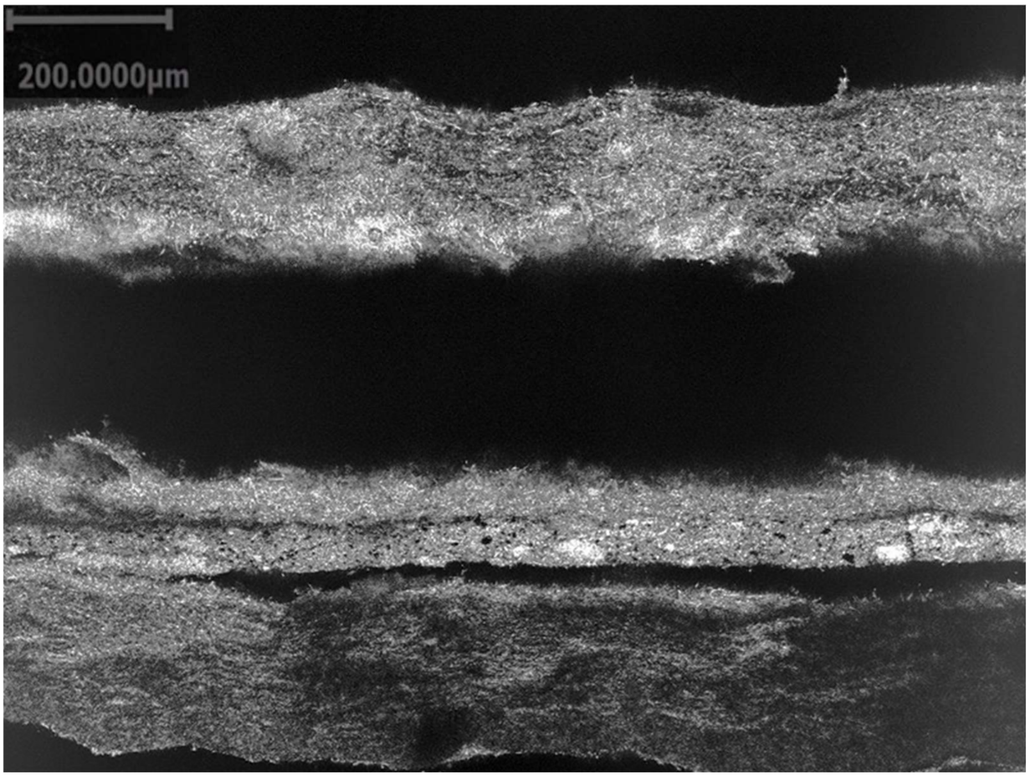

Figure S1. Cross-section laser-assisted optical microscopy showcasing the microcavity in between the two buckypapers of the electrode.

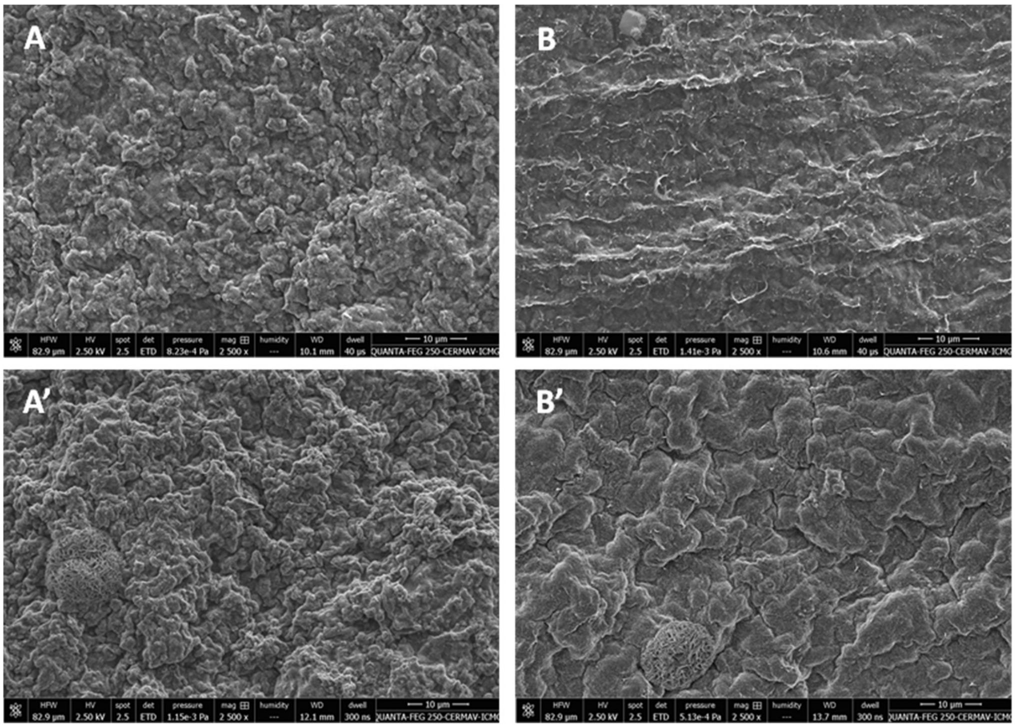

Figure S2. A and B SEM images depict the electrodes after the buckypaper preparation. (A' and B') show the electrodes after 9 months of immersion in 0.1 M PB (pH 7). In (A and A'), the inner side of the surface that contacts enzymes and the mediator is represented, whereas (B and B') display the outer side of the surface that does not come into contact with enzymes or mediators.
